# An integrated multi-omics analysis reveals a core epigenetically-activated transcriptional network driving oncogenic signaling in acute myeloid leukemia

**DOI:** 10.1007/s12672-026-05308-2

**Published:** 2026-05-30

**Authors:** Lihua Zeng, Haohao Lei, Jingnan Bi, Guowei Run, Bizhen Yu, Linhua Ji

**Affiliations:** Department of Hematology, Huadu District People’s Hospital, No. 48 Xinhua Road, Xinhua Street, Huadu District, Guangzhou, 510800 China

**Keywords:** Acute myeloid Leukemia, Multi-omics integration, Chromatin remodeling, Epigenetic dysregulation, Systems biology

## Abstract

**Purpose:**

Acute Myeloid Leukemia (AML) is driven by complex interactions between genetic mutations and epigenetic dysregulation. While alterations in chromatin modifiers are frequent, the precise downstream transcriptional networks they enable and how these networks execute the leukemogenic program remain incompletely defined.

**Methods:**

We employed an integrative bioinformatics strategy. Transcriptomic data from GSE84881 (AML stromal cells) and GSE9476 (AML blasts) identified differentially expressed genes, refined via GeneCards and CellMarker to a 32-gene AML signature. Functional enrichment (GO/KEGG) and protein-protein interaction (PPI) network analyses followed. Core hubs were validated for spatial (single-cell t-SNE) and subtype-specific expression using the Hematologic Malignancy database. Perturbation analysis (GPSAdb2.0 BioTrigger) expanded the network, with pathway enrichment on responsive genes.

**Results:**

The 32-gene signature enriched strongly in hematopoietic differentiation and unexpectedly in cross-lineage developmental pathways (e.g., gland, epithelial development). PPI topology revealed nine hubs: AFF1, TAL1, IKZF1, GATA1, NOTCH1, BCL2, IL1B, IRF4, ZAP70. Single-cell t-SNE showed distinct, non-overlapping localization patterns among AML subpopulations; box plots demonstrated marked expression heterogeneity across 26 molecular subtypes. Perturbation of these hubs generated a 500-gene set whose KEGG enrichment highlighted three interconnected layers: (i) Polycomb repression and ATP-dependent chromatin remodeling (epigenetic gatekeepers), (ii) FoxO signaling, cell cycle, and senescence (core oncogenic pathways), and (iii) broad cancer hallmarks including endocrine resistance and diverse solid tumor pathways.

**Conclusion:**

We propose a hierarchical pathomechanism: synergistic dysfunction in chromatin remodeling and Polycomb-mediated repression establishes a permissive epigenomic landscape, enabling activation of an oncogenic transcriptional network (centered on AFF1, TAL1, IKZF1, GATA1). This network then hijacks TP53/FoxO signaling to drive cell cycle escape, apoptosis resistance, and metabolic adaptation. Our findings unify disparate molecular lesions into a coherent axis and suggest new therapeutic nodes.

**Supplementary Information:**

The online version contains supplementary material available at 10.1007/s12672-026-05308-2.

## Introduction

Acute Myeloid Leukemia (AML) represents a molecularly heterogeneous malignancy originating from hematopoietic stem and progenitor cells, characterized by a blockade in differentiation and uncontrolled proliferation [1–3]. While comprehensive genomic profiling has cataloged recurrent somatic mutations in genes encoding signaling molecules, transcription factors, and epigenetic modifiers, the precise hierarchy and functional integration of these diverse lesions into a unified oncogenic program remain incompletely defined [4–6]. A critical gap in understanding lies at the interface between epigenetic dysregulation and the downstream transcriptional circuitry. Emerging evidence positions aberrations in chromatin architecture as central drivers in AML pathogenesis. Specifically, mutations or functional dysregulation affecting ATP-dependent chromatin remodeling complexes and the Polycomb Repressive Complex 2 can lead to a profound reconfiguration of the epigenomic landscape [7, 8]. This altered chromatin state is hypothesized to create a permissive environment for the ectopic activation of oncogenic transcriptional networks, yet the identity and functional coordination of the key transcription factors within these networks are not fully elucidated.

This study aims to bridge this gap by systematically identifying and characterizing a core transcriptional network activated in AML and defining its mechanistic link to upstream epigenetic alterations and downstream phenotypic consequences. Through an integrated bioinformatics approach employing multi-cohort transcriptomic analysis, stringent functional annotation, and network-based inference, we delineated a compact, high-confidence gene signature and subsequently identified nine central hub genes. These hubs encode master transcription factors and signaling regulators previously implicated in hematopoiesis and leukemia. Furthermore, by employing perturbation-based network expansion and spatial transcriptomic validation, we mapped the downstream pathways governed by this network. Our findings support a cohesive pathogenic model wherein synergistic dysfunction in chromatin remodeling and Polycomb-mediated repression enables the activation of a specific oncogenic transcriptional network, which subsequently drives leukemogenesis by subverting critical signaling hubs, including TP53 and FoxO pathways, thereby promoting hallmark cancer phenotypes.

## Methods

### Data acquisition and preprocessing

Gene expression profiles were obtained from the Gene Expression Omnibus (GEO) repository. The primary analysis was performed on dataset GSE84881, which examines transcriptional alterations in bone marrow-derived mesenchymal stromal cells from patients with Acute Myeloid Leukemia (AML) (Platform: GPL570, HG-U133_Plus_2 Array). An independent dataset, GSE9476 (Platform: GPL96, HG-U133A Array), focusing on expression changes in AML, was employed for subsequent validation. Raw CEL files were processed using the affy package in R. Normalization was conducted via the Robust Multi-array Average (RMA) algorithm, encompassing background adjustment, quantile normalization, and final expression summarization. Probes were mapped to official gene symbols using platform-specific annotation files. For genes represented by multiple probesets, the probeset with the highest median expression was retained.

### Identification of AML-associated differentially expressed genes (DEGs)

Differential expression analysis between AML and control samples within the GSE84881 training set was carried out with the limma package. Statistically significant DEGs were defined by an adjusted p-value (Benjamini-Hochberg) < 0.05 and an absolute log2 fold-change (|log2FC|) > 1. To enhance biological relevance, this initial DEG list was intersected with genes curated from two complementary resources: the GeneCards database (filtered for “Acute Myeloid Leukemia” with a relevance score > 40) and the Human Cell Marker database. This integrative filtering yielded a refined set of 32 high-confidence, AML-associated genes for downstream functional characterization.

### Functional and pathway enrichment analysis

To interpret the biological roles of the 32 candidate genes, comprehensive functional enrichment analysis was performed. Gene Ontology (GO) terms (Biological Process, Cellular Component, Molecular Function) and Kyoto Encyclopedia of Genes and Genomes (KEGG) pathways were analyzed. This was conducted using both the Metascape web-based platform with default parameters and the ShinyGO 0.85.1 web tool. Significantly enriched terms and pathways were identified based on a hypergeometric test with a False Discovery Rate (FDR) threshold of < 0.05. Results from both tools were cross-referenced for robustness.

### Protein-protein interaction (PPI) network construction and core hub gene selection

A PPI network for the 32-gene set was constructed using the STRING database (v11.5), with a medium confidence interaction score threshold (> 0.400). The network was imported into Cytoscape software (v3.9.1) for visualization and topological analysis. Key centrality metrics, including degree and betweenness centrality, were calculated using the CytoNCA and NetworkAnalyzer plugins. Genes consistently ranking high across these topological measures and supported by existing literature on leukemogenesis were prioritized as hub genes. This process identified nine core genes: AFF1, TAL1, IKZF1, GATA1, NOTCH1, BCL2, IL1B, IRF4, and ZAP70.

### Expression validation of core hub genes

To independently validate the association of the nine hub genes with AML pathology, their expression patterns and spatial distribution were interrogated using the Hematologie Malignancy database, which provides transcriptomic data across various AML subtypes and cellular contexts.

### Perturbation-based network expansion and pathway analysis

To explore the broader functional landscape downstream of the identified hub genes, a gene perturbation analysis was conducted. Using the BioTrigger module within the GPSAdb2.0 database, a list of 500 genes experimentally documented to be responsive to perturbations of the nine core hub genes was systematically compiled. This expanded gene list was subsequently subjected to KEGG pathway enrichment analysis using the clusterProfiler package in R (FDR < 0.05) to identify overarching biological mechanisms and signaling pathways potentially driven by the hub gene network in AML.

### Integrative mechanistic synthesis

Findings from all analytical stages—including differential expression, functional enrichment, PPI network topology, independent validation, and perturbation-based pathway mapping—were synthesized. This integrative interpretation, grounded in established principles of cancer biology, was performed to propose a coherent model outlining the potential collaborative roles of transcriptional dysregulation, altered signaling cascades, and epigenetic modifications in AML pathogenesis.

## Results

### Identification and functional profiling of an AML-associated gene signature

**Fig. 1:**
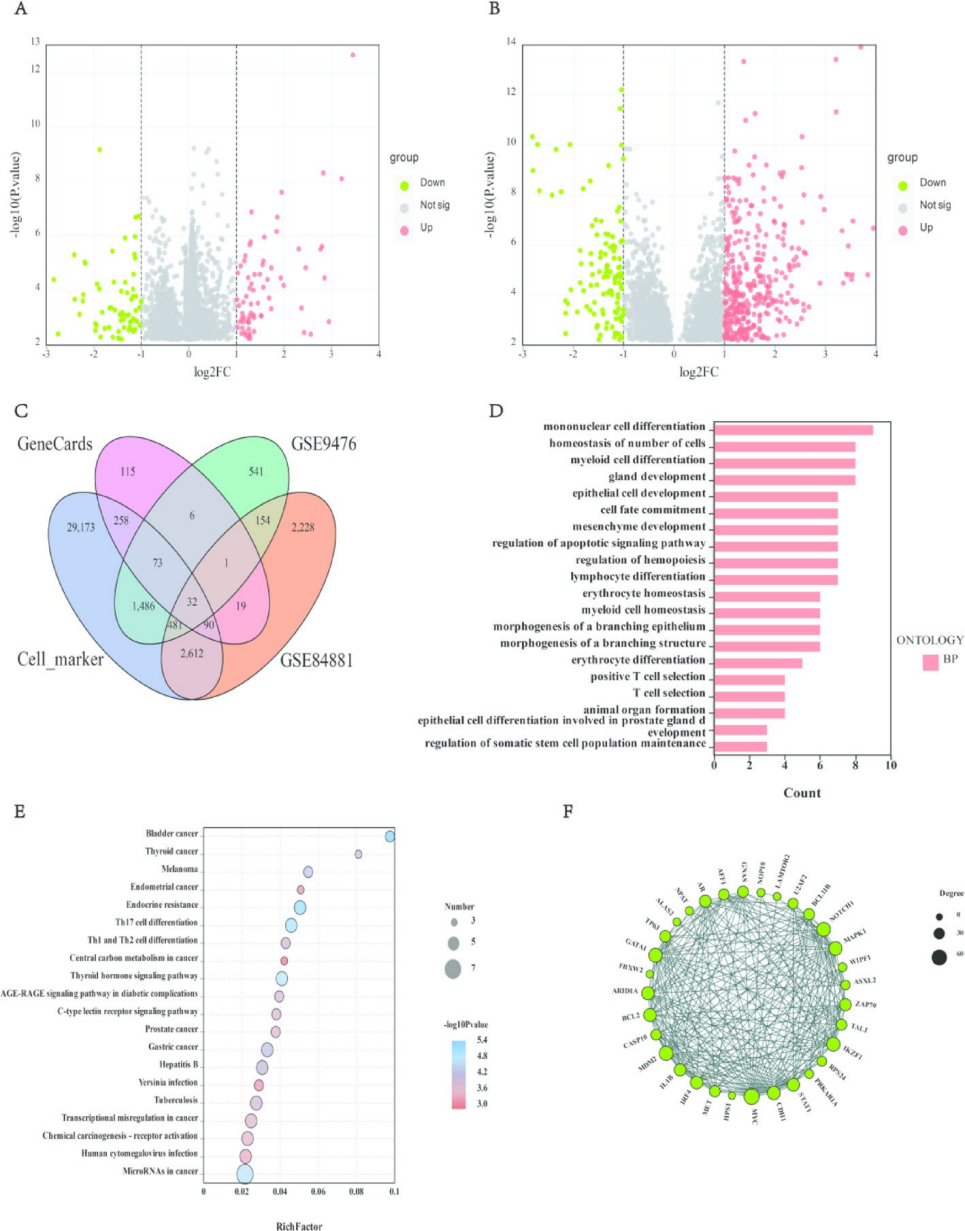
Identification and functional characterization of an AML‑associated gene signature. **A** Volcano plot showing differentially expressed genes in the GSE84881 discovery cohort (AML bone marrow mesenchymal stromal cells versus normal controls). Significantly upregulated genes (adj. *p* < 0.05, |log2FC| > 1) are shown in red, significantly downregulated genes in blue. **B** Volcano plot of differential expression in the independent validation cohort GSE9476 (AML blasts versus controls). **C** Venn diagram illustrating the intersection of DEGs from GSE84881 with high-confidence AML genes from GeneCards (relevance score > 40) and the CellMarker Human database, yielding a final 32-gene signature. **D** GO enrichment bubble plot for the 32-gene set (Biological Process category). Bubble size represents the number of genes enriched in each term; color indicates statistical significance (-log10(FDR)). Top enriched terms include “mononuclear cell differentiation”, “homeostasis of number of cells”, “myeloid cell differentiation”, “gland development”, “epithelial cell development”, and “mesenchyme development”. **E** KEGG pathway enrichment bar plot for the 32‑gene signature, showing the top 10 pathways by -log10(FDR). Significantly enriched pathways include “Transcriptional misregulation in cancer”, “Pathways in cancer”, “Hematopoietic cell lineage”, and “FoxO signaling pathway”. **F** Protein-protein interaction (PPI) network of the 32 genes constructed using the STRING database (confidence score > 0.400). Nodes are colored by degree of connectivity (darker red indicates higher degree). The nine core hub genes (AFF1, TAL1, IKZF1, GATA1, NOTCH1, BCL2, IL1B, IRF4, ZAP70) are labeled

Our analytical pipeline commenced with a comprehensive differential expression analysis. In the GSE84881 discovery cohort (AML BM-MSCs vs. controls), a standard significance threshold (adj. *p* < 0.05, |log2FC| > 1) identified a broad set of differentially expressed genes (Fig. 1A). To distill a more specific, disease-relevant signature, we integrated this list with curated genes from the GeneCards (score > 40 for AML) and CellMarker Human databases. A Venn intersection yielded a refined, high-confidence set of 32 AML-associated genes for downstream analysis (Fig. 1C). Validation in the independent GSE9476 cohort (AML blasts) confirmed a significant overlap in dysregulated genes, affirming the robustness of our signature (Fig. 1B).

To decipher the collective biological role of these 32 genes, we performed systematic enrichment analyses. Gene Ontology (GO) annotation revealed significant enrichment in terms related to hematopoietic differentiation (e.g., mononuclear and myeloid cell differentiation), cell number homeostasis, and unexpectedly, cross-lineage developmental processes such as gland and epithelial development (Fig. 1D). Notably, KEGG pathway analysis positioned this gene signature squarely within cancer biology, with the most significant enrichment in “Transcriptional misregulation in cancer” and “Pathways in cancer.” Other top-ranked pathways included “Hematopoietic cell lineage” and “FoxO signaling pathway” (Fig. 1E), implicating the signature in core oncogenic transcriptional and cell fate control circuits perturbed in AML.

A protein-protein interaction (PPI) network constructed from the 32 genes exhibited significant interconnectivity, suggesting functional cooperation (Fig. 1F). Topological analysis highlighted several central hubs. Integrating this network centrality with prior biological knowledge, we focused subsequent investigations on nine core genes: AFF1, TAL1, IKZF1, GATA1, NOTCH1, BCL2, IL1B, IRF4, and ZAP70. These encode master transcription factors (AFF1, TAL1, IKZF1, GATA1, IRF4), key signaling mediators (NOTCH1, ZAP70), and critical regulators of apoptosis (BCL2) and inflammation (IL1B).

### Spatial and subtype-specific expression patterns of core genes

**Fig. 2:**
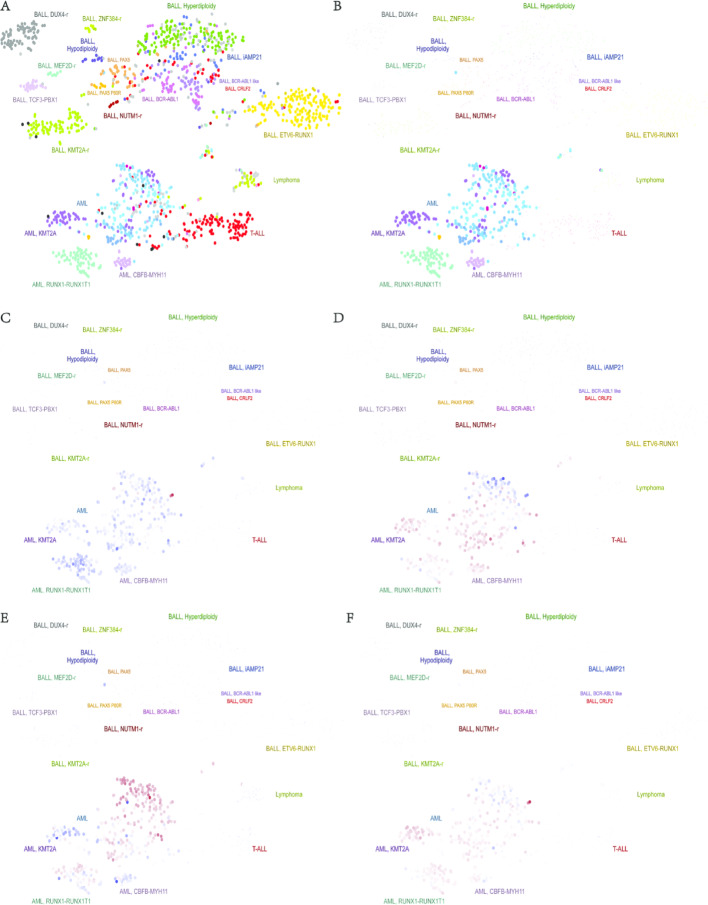

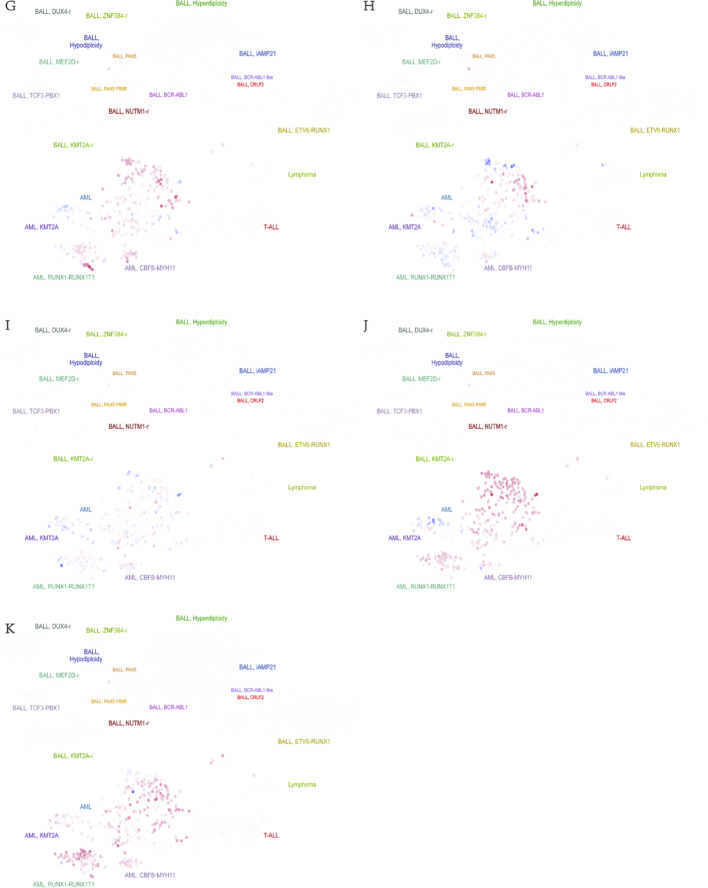
Single-cell transcriptomic distribution of core hub genes in AML. **A** t-SNE projection of all hematopoietic cells from the Hematologic Malignancy (HM) database, providing an overview of the global cellular landscape. **B** t-SNE plot of the AML compartment from the same dataset, highlighting the leukemic cell populations. **C**–**K** t-SNE maps showing the expression distribution of the nine core hub genes (C: AFF1, D: BCL2, E: GATA1, F: IKZF1, G: IL1B, H: IRF4, I: NOTCH1, J: TAL1, K: ZAP70) within AML single cells. Color intensity from blue (low) to red (high) indicates normalized expression levels. Distinct, non-overlapping localization patterns are observed, e.g., GATA1 enriched in a megakaryocytic-erythroid cluster, TAL1 broadly expressed in primitive blast populations, and IL1B localized to a putative tumor-associated macrophage/myeloid‑derived suppressor cell niche

We next leveraged single-cell transcriptomic data to contextualize the expression of these nine core genes within the AML bone marrow niche. Analysis of the Hematologie Malignancy database revealed distinct t-SNE distribution patterns for each gene across AML cell populations (Fig. 2A-K). For instance, transcription factors like GATA1 and TAL1 showed enriched expression in defined leukemic foci (e.g., a megakaryocytic-erythroid cluster for GATA1, primitive blast populations for TAL1), while IL1B exhibited a broader stromal-immune interface distribution. This single-cell heterogeneity suggests specialized, non-redundant roles within the tumor microenvironment.

**Fig. 3:**
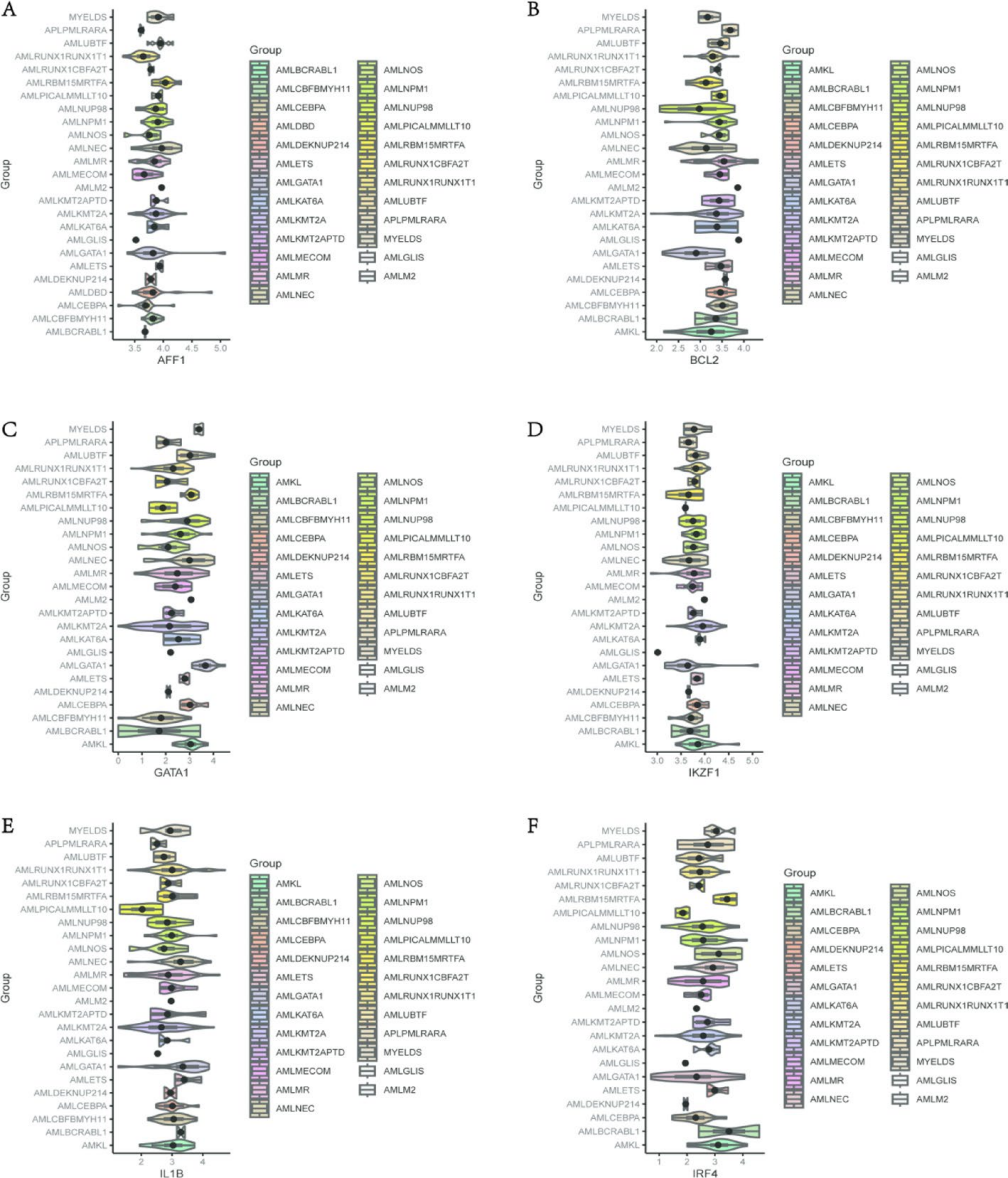

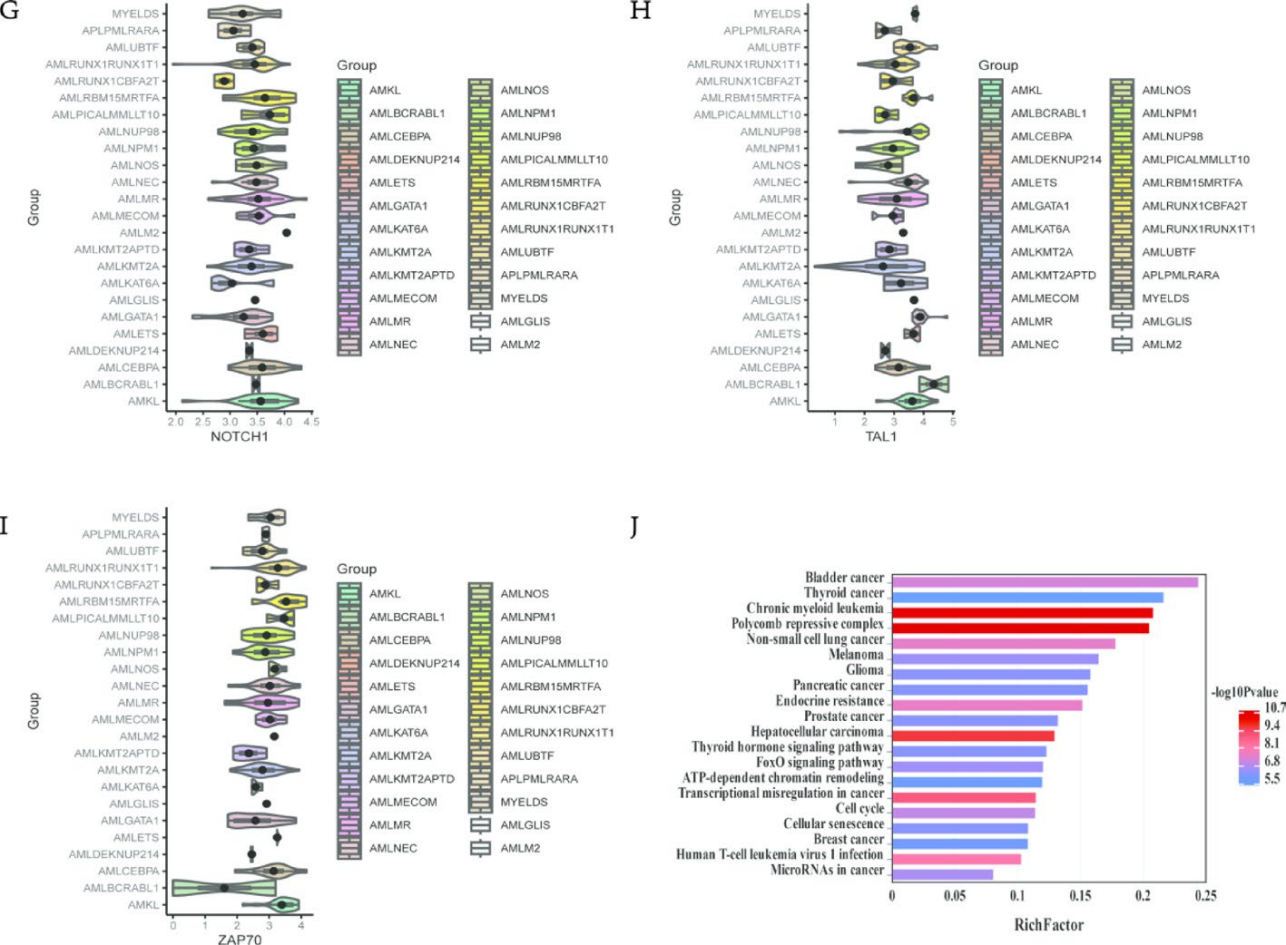
Subtype-specific expression of core hub genes and KEGG enrichment of the perturbation-expanded network. **A**–**I** Box plots showing expression levels of the nine core hub genes across 26 molecular and cytogenetic AML subtypes. The x-axis labels indicate the following subtypes: AMKL, AML_BCR-ABL1, AML_CBFB-MYH11, AML_CEBPA, AML_DEK-NUP214, AML_ETS, AML_GATA1, AML_GLIS, AML_KAT6A, AML_KMT2A, AML_KMT2A-PTD, AML_M2, AML_MECOM, AML_MR, AML_NEC, AML_NOS, AML_NPM1, AML_NUP98, AML_PICALM-MLLT10, AML_RBM15-MRTFA, AML_RUNX1-CBFA2T, AML_RUNX1-RUNX1T1, AML_UBTF, APL_PML-RARA, MYELDS. Each dot represents an individual sample; red horizontal lines indicate the median and interquartile range. Notable patterns: GATA1 peaks exclusively in AMKL and AML_GATA1; BCL2 and NOTCH1 are elevated in core-binding factor leukemias and KMT2A-PTD/NPM1 subtypes, respectively; IKZF1 is highest in BCR-ABL1-like and myelodysplasia-related AML; IRF4 is enriched in GLIS and ETS rearrangements. **J** Bubble plot of KEGG pathway enrichment analysis performed on the 500-gene perturbation set derived from the nine hub genes (GPSAdb2.0 BioTrigger module). Bubble size represents RichFactor (the ratio of enriched gene count to total gene count in the pathway). Color scale indicates statistical significance (-log10(FDR)). Key pathways are grouped into three mechanistic layers: Epigenetic regulation (Polycomb repressive complex, ATP-dependent chromatin remodeling), Core oncogenic signaling (FoxO signaling pathway, Cell cycle, Cellular senescence, Chronic myeloid leukemia, Transcriptional misregulation in cancer), and Broad cancer hallmarks (Thyroid hormone signaling, Endocrine resistance, and various solid tumor pathways such as bladder, lung, pancreatic, breast cancer, etc.)

Furthermore, examining their expression across molecular and cytogenetic AML subtypes uncovered pronounced variability (Fig. 3A-I). GATA1 peaked exclusively in the AMKL subtype and AML-GATA1-annotated cases; BCL2 and NOTCH1 displayed elevated expression in core-binding factor leukemias and KMT2A-PTD/NPM1 subtypes, respectively, both associated with adverse prognosis. Meanwhile, IKZF1 and IRF4 showed distinct patterns in BCR-ABL1-like and GLIS/ETS rearrangement subtypes, respectively. This subtype-specific dysregulation reinforces the functional importance of these hubs across the disease spectrum.

### Perturbation-based network expansion implicates epigenetic and signaling hubs

To uncover the downstream consequences and broader regulatory network governed by the nine core genes, we performed a gene perturbation analysis. Using the GPSAdb2.0 BioTrigger module, we generated a list of 500 genes consistently reported to respond to perturbations (knockdown/overexpression) of our core hubs. KEGG enrichment analysis of this expanded network was highly revealing (Fig. 3J). We observed significant enrichment for specific, interlinked mechanistic modules: Epigenetic Dysregulation: “Polycomb repressive complex (PRC)” and “ATP-dependent chromatin remodeling” pathways were prominently enriched. Oncogenic Signaling: Key pathways included “FoxO signaling pathway,” “Cell cycle,” and “Cellular senescence.” Disease Context: Notable enrichment for “Chronic myeloid leukemia” and “Transcriptional misregulation in cancer” confirmed the network’s direct relevance to hematopoietic malignancies.

### An integrative model of coordinated dysregulation in AML

Based on the multi-omics evidence presented—spanning transcriptional profiling, network topology, single-cell mapping, and perturbation-derived pathway enrichment—we propose an integrated pathogenic model for AML. Our findings indicate that synergistic dysfunction of chromatin remodeling machinery and the Polycomb Repressive Complex precipitates a profound reorganization of the epigenetic architecture in AML progenitor cells. This reconfigured epigenomic state enables sustained de-repression and consequent hyperactivation of a potent oncogenic transcriptional network, whose core regulatory nodes include the master transcription factors AFF1, TAL1, IKZF1, and GATA1. The pathological output of this activated network is subsequently channeled through pivotal signaling hubs, including the TP53 and FoxO pathways. This cascade of dysregulation fundamentally reprograms cellular homeostasis, driving the acquisition of core leukemogenic phenotypes: loss of cell cycle control, resistance to apoptosis, and profound metabolic adaptation—collectively underpinning disease initiation and progression.

## Discussion

The findings presented herein integrate multiple analytical dimensions to propose a coherent, hierarchical model of AML pathogenesis. The initial computational pipeline, synthesizing differential expression data from stromal and leukemic compartments with established disease-gene databases, yielded a focused 32-gene signature. This signature was not randomly assembled but was demonstrably enriched for processes central to hematopoietic differentiation and, unexpectedly, cross-lineage developmental programs—a finding that highlights the ectopic activation of stem-cell-like transcriptional circuitry in AML. The strong enrichment for pathways such as “Transcriptional misregulation in cancer” and “Hematopoietic cell lineage” underscores the dual importance of cell-autonomous transcriptional dysregulation and lineage-specific context in AML [9–10].

The subsequent network topology analysis revealed that this signature is organized around a set of highly interconnected hub genes. The identification of AFF1, TAL1, IKZF1, and GATA1 as core transcriptional regulators within this network is highly significant. These factors are not merely co-expressed but are known to participate in critical, often aberrant, regulatory circuits during hematopoiesis and leukemogenesis [11–15]. Their co-localization as central nodes in our derived network suggests a potential for functional cooperation or synergistic dysregulation. The single-cell and subtype-specific expression patterns further contextualize their roles, indicating that this network’s activity is not uniform but is modulated by the cellular and genetic context of the leukemia [Figs. 2 and 3A-I].

A pivotal insight from this study is the proposed mechanistic link between upstream epigenetic alterations and the activation of this transcriptional network. The perturbation-based analysis provided critical data supporting this connection. The significant enrichment of “Polycomb repressive complex” and “ATP-dependent chromatin remodeling” pathways among genes responsive to hub gene perturbations strongly implies a bidirectional or reinforcing relationship [Fig. 3J]. This aligns with a growing body of literature suggesting that the functional impairment of complexes such as PRC2, often mediated by EZH2 dysregulation, and the BAF chromatin remodeling complex, involving SMARCA4, can lead to widespread epigenetic derepression [16–20]. Our model posits that this derepression specifically facilitates the binding and activity of the identified master transcription factor network, effectively translating epigenetic lesions into a coordinated transcriptional output.

This activated network, in turn, executes its oncogenic program by impinging on fundamental signaling and regulatory pathways. The robust enrichment of the “FoxO signaling pathway” and “Cell cycle” among the perturbation-responsive genes is particularly noteworthy [Fig. 3J]. FoxO transcription factors are key integrators of cellular metabolism, stress response, and survival signals, and their aberrant regulation is a common feature in therapy-resistant AML [21–22]. Similarly, the inferred connection to TP53 pathway dysfunction, a hallmark of high-risk disease, provides a plausible mechanism for how this network promotes genomic instability and apoptotic evasion [23]. Thus, the core transcriptional network acts as a critical intermediary, conveying the permissive signal from the disrupted epigenetic landscape to the effector machinery that directly controls cell fate decisions.

Several limitations of this study warrant consideration. The primary evidence is derived from computational analyses of publicly available datasets, and the proposed model requires direct experimental validation. Future work should employ genetic and pharmacological approaches in relevant AML models to functionally test the necessity and sufficiency of the identified hub genes and to dissect the causality within the proposed epigenetic-transcriptional-signaling axis. Based on this model, the investigation into potential synergistic therapeutic strategies holds significant promise. Specifically, exploring whether pharmacological agents that target critical downstream effectors—such as specific cell cycle kinases—can enhance the efficacy of therapies aimed at the upstream epigenetic machinery represents a logical and compelling avenue for future translational research.

## Conclusion

In summary, this integrative systems-level analysis delineates a pathogenic framework for AML that connects discrete molecular lesions to a unified oncogenic phenotype. We demonstrate that a set of nine core transcriptional and signaling regulators forms a highly interconnected network aberrantly active in AML. Our data support a model wherein primary defects in chromatin remodeling and Polycomb-mediated silencing converge to establish an epigenomic landscape permissive for the activation of this network. Once engaged, this transcriptional hub orchestrates leukemogenesis by dysregulating pivotal downstream pathways, including FoxO signaling, cell cycle control, and cellular senescence—thereby enforcing the hallmarks of cancer. This study not only elucidates a novel mechanistic axis in AML but also nominates specific nodes within this hierarchy as potential targets for therapeutic intervention, particularly in the context of combinatorial strategies aimed at multiple layers of dysregulation.

## Supplementary Information

Below is the link to the electronic supplementary material.

Supplementary Material 1.

Supplementary Material 2.

Supplementary Material 3.

## Data Availability

The datasets analysed during the current study are available in the Gene Expression Omnibus (GEO) repository, under accession numbers GSE84881 and GSE9476. All data generated or analysed during this study are included in this published article.

## References

[1] Russo S, Fazio M, Mirabile G, et al. Bone Marrow Edema and Tyrosine Kinase Inhibitors Treatment in Chronic Myeloid Leukemia. Diagnostics (Basel). 2025;15(24):3112. 41464113 10.3390/diagnostics15243112 PMC12731681

[2] Thakur RK, Wang ES. The promise of menin inhibitors: from approval to triplet regimens. Hematol Am Soc Hematol Educ Program. 2025;2025(1):599–606. 10.1182/hematology.2025000755 PMC12891526 41347983

[3] Sun K, Zhou J, Yao H, et al. LncRNA SNHG11 expression in acute myeloid leukemia patients and its relationship with the biology of acute myeloid leukemia cells. Clin Exp Med. 2025;26(1):20. 41249677 10.1007/s10238-025-01923-5 PMC12628419

[4] Telonis AG, Stanley RF, Adelman ER, et al. Synergistic intragenic epigenetic deregulation by IDH2 and SRSF2 mutations causes mis-splicing of key transcriptional regulators. Sci Adv. 2026;12(1):eadu8292. 41481703 10.1126/sciadv.adu8292 PMC12758512

[5] Kelly LM, Rutter JC, Lin KH, et al. Targeting a lineage-specific PI3Kɣ-Akt signaling module in acute myeloid leukemia using a heterobifunctional degrader molecule. Nat Cancer. 2024;5(7):1082–101. 38816660 10.1038/s43018-024-00782-5 PMC11778622

[6] Pawlik B, Madzio J, Rydzyńska Z, et al. mTOR Modulation Affects Galectin-1 Expression in KMT2A-rearranged Acute Lymphoblastic Leukemia Cells. Anticancer Res. 2026;46(2):651–66. 41617461 10.21873/anticanres.17976

[7] Xie J, Soleimani Samarkhazan H. Beyond the DNA sequence: mapping the dynamic epigenetic landscape for risk stratification and therapeutic intervention in acute myeloid leukemia. Clin Exp Med. 2025;26(1):56. 41398141 10.1007/s10238-025-01972-w PMC12705804

[8] Yan Z, Yuan L, Wang J, et al. PRC2-Related Epigenetic Age Acceleration in Acute Myeloid Leukemia with DNMT3A and IDH2 Mutations. Adv Biol (Weinh). 2026;10(1):e00710. 41556261 10.1002/adbi.202500710 PMC12817234

[9] Daniel MM, Andwey GH. Integrative Bioinformatics Reveals Novel Molecular Mechanisms and Therapeutic Targets in Acute Myeloid Leukaemia. J Cell Mol Med. 2026;30(1):e71007. 41492215 10.1111/jcmm.71007 PMC12771679

[10] Subklewe M, Rutella S, Curti A. The immunotherapy landscape in AML: Defining knowledge gaps toward rational combinatorial strategies. Semin Hematol. 2025;62(3):209–17. 41046175 10.1053/j.seminhematol.2025.08.003

[11] O’reilly J, Lawson MJ, Riegleman K, et al. Acute Myeloid Leukemia With KMT2A Rearrangement Presenting as Skin Hyperpigmentation. J Cutan Pathol. 2026;53(3):258–62. 41351321 10.1111/cup.70032

[12] Huang J, Wang M, Zhang Z, et al. TMEM91::TAL1 Fusion gene in a middle-aged female with rapid MDS to secondary AML progression: a case report. Hematology. 2025;30(1):2594370. 41317203 10.1080/16078454.2025.2594370

[13] Ahlgren L, Pilheden M, Sturesson H, et al. The genomic landscape of relapsed infant and childhood KMT2A-rearranged acute leukemia. Nat Commun. 2025;16(1):8964. 41062506 10.1038/s41467-025-64190-8 PMC12508131

[14] Zhang L, Liu Y, Ding Y, et al. [Analysis of a child with Congenital leukemia and mosaicism trisomy 21 syndrome without GATA1 gene mutation]. Zhonghua Yi Xue Yi Chuan Xue Za Zhi. 2025;42(6):751–5. 40763975 10.3760/cma.j.cn511374-20250611-00359

[15] Mehra AT, Wojcik MH. Presentation and Longer-Term Outcomes in Mosaic Trisomy 21 Causing Isolated Transient Abnormal Myelopoiesis. Am J Med Genet A. 2025;197(6):e63979. 39995092 10.1002/ajmg.a.63979

[16] Verma S, Goyal N, Goyal S, et al. EZH2 Dysregulation and Its Oncogenic Role in Human Cancers. Cancers (Basel). 2025;17(19):3111. 41097640 10.3390/cancers17193111 PMC12523533

[17] Tang M, Gong M, Liu X, et al. Recent update on the development of EZH2 inhibitors and degraders for cancer therapy. Eur J Med Chem. 2025;299(0):118106. 40884958 10.1016/j.ejmech.2025.118106

[18] Wang X, Wang Y, Xie M, et al. Hypermethylation of CDKN2A CpG island drives resistance to PRC2 inhibitors in SWI/SNF loss-of-function tumors. Cell Death Dis. 2024;15(11):794. 39500892 10.1038/s41419-024-07109-3 PMC11538500

[19] Wang J, Yang L, Du Y, et al. BRG1 programs PRC2-complex repression and controls oligodendrocyte differentiation and remyelination. J Cell Biol. 2024;223(7):e202310143. 38652118 10.1083/jcb.202310143 PMC11040499

[20] Keller PJ, Adams EJ, Wu R, et al. Comprehensive Target Engagement by the EZH2 Inhibitor Tulmimetostat Allows for Targeting of ARID1A Mutant Cancers. Cancer Res. 2024;84(15):2501–17. 38833522 10.1158/0008-5472.CAN-24-0398 PMC11292196

[21] Zhang X, Yang J, Wen Y, et al. [METTL3-mediated m(6)A modification promotes FOXO3 expression and anthracycline resistance in acute myeloid leukemia cells through autophagy regulation]. Nan Fang Yi Ke Da Xue Xue Bao. 2025;45(3):470–8. 40159961 10.12122/j.issn.1673-4254.2025.03.04 PMC11955884

[22] Reichelt P, Bernhart S, Platzbecker U, et al. MicroRNA Screening Reveals Upregulation of FoxO-Signaling in Relapsed Acute Myeloid Leukemia Patients. Genes (Basel). 2024;15(12):1625. 39766892 10.3390/genes15121625 PMC11675194

[23] Olesinski EA, Bhatt S. Pandora’s Box of AML: How TP53 Mutations Defy Therapy and Hint at New Hope. Biomedicines. 2025;13(12):3007. 41463019 10.3390/biomedicines13123007 PMC12731087

